# Antinociceptive and anti-inflammatory activities of the *Jatropha isabellei* dichloromethane fraction and isolation and quantitative determination of jatrophone by UFLC-DAD

**DOI:** 10.1080/13880209.2017.1295999

**Published:** 2017-03-01

**Authors:** Janaina Kieling Fröhlich, Taciane Stein, Layzon Antônio da Silva, Maique Weber Biavatti, Carlos Rogério Tonussi, Elenara Lemos-Senna

**Affiliations:** a Department of Pharmaceutical Sciences, Federal University of Santa Catarina, Campus Trindade, Florianópolis, SC, Brazil;; b Department of Pharmacology, Federal University of Santa Catarina, Campus Trindade, Florianópolis, SC, Brazil

**Keywords:** Arthritis, inflammation, pain

## Abstract

**Context:***Jatropha isabellei* Müll. Arg. (Euphorbiaceae) has been used in the traditional medicine to treat arthritis.

**Objective:** To evaluate the anti-inflammatory and antinociceptive activities of the dichloromethane fraction (DF*_Ji_*) from underground parts of *J. isabellei*, and to develop an analytical method to quantify the diterpene jatrophone.

**Materials and methods:** Anti-inflammatory and antinociceptive activities of the DF*_ji_* were determined by an acute arthritis model through assessment of the paw elevation time (PET) and articular diameter (AD) of Wistar rats treated orally (50, 100 or 200 mg/kg in a single-dose), and intravenously (0.1, 1, 10, 25 or 50 mg/kg in a bolus administration). The isolation of jatrophone from the DF*_ji_* was carried out and confirmed by spectroscopic techniques. A UFLC-DAD method was developed and validated.

**Results:** When orally administered, the highest dose (200 mg/kg) of DF*_Ji_* was able to significantly reduce the PET to 24.8 ± 1.4 s (*p* < 0.01), when compared with the control group (33.7 ± 1.8 s). The administration of the intravenous dose of 10 mg/kg reduced the PET to 14.8 ± 0.3 s (*p* < 0.001). The oral and intravenous administration of the DF*_Ji_* at dose of 200 and 10 mg/kg significantly prevented the formation of edema, reducing the AD in 25.3% and 32.5% (*p* < 0.01), respectively. The UFLC-DAD method allowed the quantification of jatrophone, which was found to be around 90 μg/mg of fraction.

**Discussion and conclusion:** The DF*_Ji_* displayed antinociceptive and antiedematogenic activities, representing a promising plant product for the arthritis treatment.

## Introduction

Arthritis is a disabling condition that affects millions of people worldwide. It is a form of joint disorder that involves inflammation of one or more joints with infiltration of inflammatory cells, synovial hyperplasia, cartilage destruction, bone erosion, narrowing of the joint space and ankylosis of the joint (Bendele et al. 1999). The major complaint by individuals who have arthritis is joint pain, which is due to an inflammation that occurs around the joint, and is associated with muscle weakness, loss of flexibility, joint stiffness, fatigue, loss of quality of life, etc. (Lee 2013). The most common forms of arthritic conditions are osteoarthritis, rheumatoid arthritis, and gout, in which the gout is responsible for the worst episodes of acute pain and can lead to the development of chronic and tophaceous gouty arthritis, and renal damage (Cannella & Mikuls 2005). There is no available cure for these arthritic conditions and the treatment options may include physical therapy, lifestyle changes (including exercise and weight control) and medications. The pharmacological treatment for arthritis includes the administration of analgesics, corticosteroids, disease-modifying antirheumatic drugs (DMARDs), non-steroidal anti-inflammatory drugs (NSAIDs) and biological drugs (Negrei et al. 2016). However, besides presenting several adverse effects, many patients are refractory to these drugs, making medication adherence difficult. These drawbacks have stimulated research on the arthritis treatment and plant constituents (Rates 2001; Khanna et al. 2007; Ghosh et al. 2016).

*Jatropha isabellei* Müll. Arg. (Euphorbiaceae) is a shrub with red-violet inflorescences known in the Paraguayan and Brazilian folk medicine as ‘yagua rova’, ‘turubiti’ and ‘mamoneiro do campo’ (Basualdo et al. 1991; Riveros et al. 2009; Fröhlich et al. 2013). The infusion or decoction obtained from the underground parts of *J. isabellei* has been popularly used to treat different types of arthritis (Basualdo et al. 1991). In fact, the antinociceptive and anti-inflammatory properties of the crude extract were evidenced in a rat gout model induced by sodium monourate (MSU) crystals. Although the crude extract was able to prevent the mechanical allodynia, thermal hyperalgesia, edema, and neutrophil infiltration induced by intra-articular MSU injection, it was not able to alter the uric acid levels increased by potassium oxanate (Silva et al. 2013). Also, the crude extract of *J. isabellei* was able to inhibit the xanthine oxidase activity *in vitro* only at high concentrations. These results suggested that neither the xanthine oxidase inhibition nor the decrease of uric acid blood levels are implicated in the antinociceptive and antiedematogenic effects verified for *J. isabellei* (Silva et al. 2013).

Many studies have been carried out to elucidate the chemical composition of the underground parts of *J. isabellei*. The combined petroleum ether and ethyl acetate extracts obtained from rhizomes of this plant have been found to include triterpene acetyl aleuritolic acid, sesquiterpene cyperenoic acid, and diterpenes jatrophone and jatropholones A and B, besides a monoterpene and a firstly related diterpene named 9β,13α-dihydroxyisabellione (Pertino et al. 2007). Also, acetyl aleuritolic acid and a binary mixture of sitosterol-3-*O*-β-d-glucoside and stigmasterol were identified from the dichloromethane fraction obtained from the underground parts of this plant (Fröhlich et al. 2013).

In general, terpenes are recognized for having anti-inflammatory and analgesic properties, and therefore have been considered as potential candidates for new drugs intended to control painful syndromes and inflammatory diseases (Sultana & Saify 2012; Guimarães et al. 2014). Since nonpolar bioactive compounds as terpenes and steroids can be extracted using dichloromethane as solvent, and given the popular use of this plant to treat arthritis, the aim of this study was to evaluate the anti-inflammatory and analgesic activities of the dichloromethane fraction after oral and intravenous administration in an acute arthritis model induced by carrageenan in rats. Additionally, considering that no analytical methods have been reported to chemically characterize the *J. isabellei* dichloromethane fraction, an important constituent of this fraction was identified and isolated, and an analytical methodology of ultra-fast liquid chromatography with diode array detection (UFLC-DAD) was developed and validated to quantitatively determine this compound.

## Materials and methods

### Plant collection and extraction

*Jatropha isabellei* was collected in the municipality of Cacequi (State of Rio Grande do Sul, Brazil, coordinates: latitude 29°53′01″ S and longitude 54°49′30″ W) in May of 2008. The plant material was identified by the botanist Prof. Renato A. Záchia (Federal University of Santa Maria) and an exsiccate was archived in the herbarium of the Biology Department at the Federal University of Santa Maria (SMDB 11816). The underground parts were dried at room temperature and powdered in a knife mill. The powder was macerated with 70% (v/v) (plant to solvent ratio of 1:3, w/v) ethanol for 10 days at room temperature. After filtration, the ethanol was evaporated under reduced pressure and this dispersion was partitioned with dichloromethane to obtain its respective fraction, which was further taken to dryness under reduced pressure, resulting in the dichloromethane fraction (DF*_Ji_*, yield 3.7%).

### Drugs and reagents

The dichloromethane fraction from *J. isabellei* (DF*_Ji_*) was resuspended in a mixture composed of dimethyl sulphoxide (DMSO), polyethylene glycol 400 (PEG 400), and phosphate buffered saline (PBS), pH 7.4 (5:47.5:47.5 v/v) for oral administration (p.o.) in rats. For intravenous administration, the fraction was dissolved in a mixture composed of DMSO, polysorbate 80, and saline solution (5:4:91 v/v). A combination of κ- and λ-carrageenan was purchased from BDH Chemicals Ltd. (London, UK). Dexamethasone and indomethacin were purchased from Deg (São Paulo, Brazil), and colchicine from Sigma-Aldrich (St Louis, MO). The jatrophone isolation from the dichloromethane fraction required the use of Silica Gel 60 and Silica Gel 60 F254-coated plates, which were purchased from Merck (Darmstadt, Germany). Analytical grades hexane, acetone, dichloromethane, ethanol and methanol were purchased from Vetec (Duque de Caxias, Brazil). Acetonitrile HPLC grade was purchased from Panreac (Barcelona, Spain). The ultrapure water utilized in the UFLC analyses was obtained using a Milli-Q purification system (Millipore, Billerica, MA).

### Antinociceptive and anti-inflammatory activities in a carrageenan-induced arthritis model of the DF_Ji_

#### Animals

The experiments were conducted in accordance with the National Institutes of Health (NIH) guide for the care and use of laboratory animals (NIH, 1996), the ethical guidelines of the International Association for the Study of Pain (IASP 1983) and approved by the local committee for ethical use of animals (P00723/CEUA-UFSC). All experiments were performed using adult male Wistar rats weighing 250–300 g. The animals were housed under a controlled temperature (21 ± 2 °C) on a 12 h light/dark cycle with standard lab chow and water *ad libitum* until the experimental sessions. The animals were acclimatized into the experimental room for at least 30 min before the experiments.

#### Carrageenan-induced articular incapacitation in rats

Articular incapacitation was induced by the injection of 300 μg of carrageenan (solubilized in 50 μL sterile 0.9% saline) into the right knee joint of the rats. In this assay, the animals were stimulated to walk on a revolving steel cylinder (constant speed of 3 rpm) wearing metallic gaiters in their hind paws. The right paw gaiter was connected to a computer system that counted the total duration of no contact on the cylinder surface during the one minute test period. This paw elevation time (PET), in seconds, was taken as an estimate of nociception (Tonussi & Ferreira 1992). Two hours after the carrageenan injection, the animals were treated with the DF*_Ji_* either orally (50, 100 or 200 mg/kg) in a single-dose by gavage or intravenously (0.1, 1, 10, 25 or 50 mg/kg) in a bolus administration by gingival vein punction (Oliveira et al. 2009). The PET was evaluated in the 3^rd^ h and hourly until the 6^th^ h, and presented as an average of these time points.

Oral dexamethasone (10 mg/kg), colchicine (30 mg/kg) and indomethacin (5 mg/kg) were used as positive controls and their effects were compared with that of the dichloromethane fraction. Dexamethasone (1 mg/kg) was also used as a positive control in the intravenously treated group. Negative control groups were treated only with the vehicle.

#### Edema measurement

Articular diameter (AD) was used to quantify the inflammatory edema induced by carrageenan and it was obtained by measuring the medio-lateral axis (in mm) of the knee-joint, using a micrometer at three consecutive arbitrary points in a proximo-distal direction. Two hours after the carrageenan injection, the animals were treated with the DF*_Ji_* either orally by gavage (50, 100 or 200 mg/kg in a single-dose) or intravenously in a bolus administration by gingival vein punction (0.1, 1, 10, 25 or 50 mg/kg) (Oliveira et al. 2009). The AD measured just before the carrageenan injection was subtracted from the AD values taken hourly from the 3^rd^ to the 6^th^ h, just after the incapacitation measurement, and presented as an average of these time points. Oral dexamethasone (1 mg/kg), colchicine (30 mg/kg) and indomethacin (5 mg/kg) were used as positive controls and their effects were compared with that of the dichloromethane fraction. Negative control groups were treated only with the vehicle.

### Statistical analysis

The sample size for incapacitation and articular edema were estimated using a statistical power test, and a minimum of 6 animals were used for both parameters. Data were expressed as mean ± SEM or mean ± SD. Statistical significance between groups was calculated by one-way analysis of variance (ANOVA) followed by Dunnett’s multiple comparison test or Student’s *t*-test when appropriate. Only *p* values lower than 0.05 (*p* < 0.05) were considered significant.

### Isolation and identification of jatrophone in the DF_Ji_

The dichloromethane fraction from the underground parts of *J. isabellei* (1.05 g) was submitted to column chromatography on silica gel 60 (70 g) and eluted with different proportions of hexane:acetone (from 95:5 to 0:100) and acetone:methanol (from 90:10 to 30:70). This procedure resulted in sub-fractions that were analyzed by thin layer chromatography (TLC) and grouped based on similarity of their chromatographic profiles after revelation with sulfuric anisaldehyde/100 °C for 2 min. The characterization of the isolated compound **1** was performed by ^13^C NMR in a Bruker DPX 400 equipment and for the compound **2** the characterization was performed by 1D and 2D nuclear magnetic resonance experiments in a Bruker Ascend 600 equipment, and by HRMS (APPI-QTof) using a Bruker micrOTOF QII. The UV/VIS spectrum of the isolated compound was recorded using an SPD-M20 DAD UV/VIS detector, during the UFLC analysis.

### Determination of jatrophone in the DF_Ji_ by UFLC-DAD

#### Chromatographic conditions

The chromatographic analyses were performed on a UFLC-DAD system (Shimadzu, Tokyo, Japan) equipped with a LC-20AD binary pump, an SIL-20AC HT auto-sampler, a CTO-20A forced air-circulation-type column oven, an SPD-M20 photo diode array UV/VIS detector, and the software LC Solution 1.2 (Shimadzu, Tokyo, Japan). The analyses were carried out in reversed phase mode using a Phenomenex Luna C18 column (150 mm × 4.6 mm × 5 μm) and a mobile phase consisted of acetonitrile and water, filtered prior to use through 0.45 μm polyvinylidene flouride (PVDF) and a regenerated cellulose (RC) membrane filter, respectively. In order to determine the jatrophone concentration in the DF*_Ji_*, the mobile phase was eluted at a flow rate of 1.0 mL/min using the following gradient program: 53–65% acetonitrile from 0 to 12 min, and 65–75% acetonitrile from 12 to 15 min. After this time, reequilibration was performed to restore the system and column to the initial mobile phase condition prior to the next injection. The total runtime was 23 min. The injection volume of the samples was 20 μL and the detection of jatrophone was monitored at 280 nm, according to its maximum absorption.

#### Preparation of the samples and standard solutions

Jatrophone, identified as a possible chemical marker of the DF*_Ji_*, was used as an external standard. Working standard solutions of this diterpene were prepared by dissolving it in acetonitrile to obtain a concentration of 100 μg/mL. In order to quantitatively determine jatrophone, the DF*_Ji_* was dissolved in acetonitrile at a concentration of 0.5 mg/mL. All standard solutions and samples were filtered through a 0.45 μm PVDF membrane filter before the UFLC injection.

#### Validation of the UFLC method

The UFLC method was validated according to the International Conference on Harmonization (ICH) and the ANVISA guidance, and included the parameters of linearity, limits of detection (LOD) and quantification (LOQ), specificity, accuracy, and precision (ANVISA 2003; ICH 2005). The linearity of the analytical method was assessed by constructing calibration curves for jatrophone, in triplicate, after analyzing eight jatrophone standard solutions at concentrations ranging from 1.0 to 100.0 μg/mL, in three different days. The linearity of the method was evaluated by calculating the linear regression coefficient using the least-square method. The LOD and LOQ were determined at signal-to-noise ratios (S/N) of 3 and 10, respectively, based on the standard deviation of the *y*-intercept of the regression curves (ICH 2005). In order to determine repeatability (intra-day precision), an analysis of the DF*_Ji_* was performed in sextuplicate at one level concentration in a single day. The intermediate precision (inter-day precision) was determined through a single analysis of the DF*_Ji_* in sextuplicate for another two consecutive days. The results were expressed as relative standard deviation (%RSD). The accuracy of the method was determined through an analyte recovery test by spiking a dichloromethane fraction sample with standard solutions of jatrophone at three levels (low, medium and high). Specificity was confirmed by the peak purity index obtained directly from the spectral analysis report.

## Results

### Antinociceptive and anti-inflammatory activities of the DF_Ji_

#### Effect of oral administration of the DF_Ji_ on the rat paw elevation time

**Figure 1.**
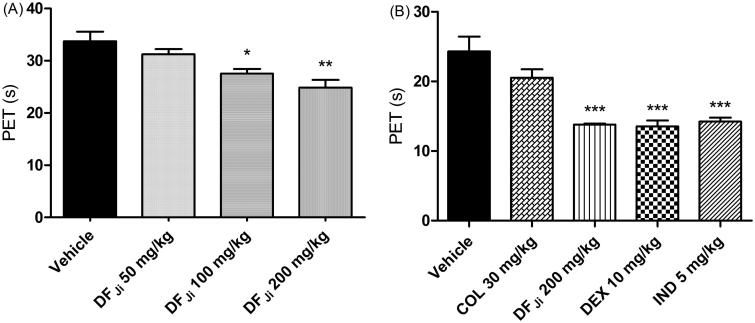
(A) Effect of the *J. isabellei* dichloromethane fraction (DF*_Ji_*) 50, 100 and 200 mg/kg on paw elevation time (PET) after oral administration. (B) Effect of oral administration of the *J. isabellei* dichloromethane fraction (200 mg/kg), indomethacin (IND, 5 mg/kg), colchicine (COL, 30 mg/kg) and dexamethasone (DEX, 10 mg/kg) on paw elevation time (PET). The animals were treated 2 h after the intra-articular carrageenan injection (300 μg/knee). The negative control group received only the vehicle (DMSO:PEG 400:PBS 5:47.5:47.5). *p < 0.05; **p < 0.01 and ***p < 0.001 represent a significant difference compared with the negative control group. The statistical analysis was performed using one-way ANOVA followed by Dunnett’s *post hoc* test.

Animals treated only with vehicle p.o., 2 h after carrageenan injection (negative control group), showed an average PET value of 33.7 ± 1.8 s. The DF*_Ji_* was administered orally at doses of 50, 100 and 200 mg/kg. The higher doses produced average PET values of 27.5 ± 0.9 s (*p* < 0.05) and 24.8 ± 1.4 s (*p* < 0.01), which were significantly different from those of the negative control group (Figure 1(A)).

The effect obtained with the administration of 200 mg/kg DF*_Ji_* on PET was then compared with three drugs commonly used for arthritis: dexamethasone (10 mg/kg), indomethacin (5 mg/kg), and colchicine (30 mg/kg) (Figure 1(B)). The incapacitation reversal produced by the DF*_Ji_* (200 mg/kg, *p* > 0.001) was similar to the effects observed with dexamethasone and indomethacin treatments. Colchicine did not reduce the PET under these conditions. The average PET values were 24.3 ± 2.1, 13.8 ± 0.1, 13.5 ± 0.8, 14.2 ± 0.5 and 20.5 ± 1.2 s, for vehicle, dichloromethane fraction, dexamethasone, indomethacin and colchicine, respectively (Figure 1(B)).

#### Effect of intravenous administration of DF_Ji_ on the rat paw elevation time

**Figure 2.**
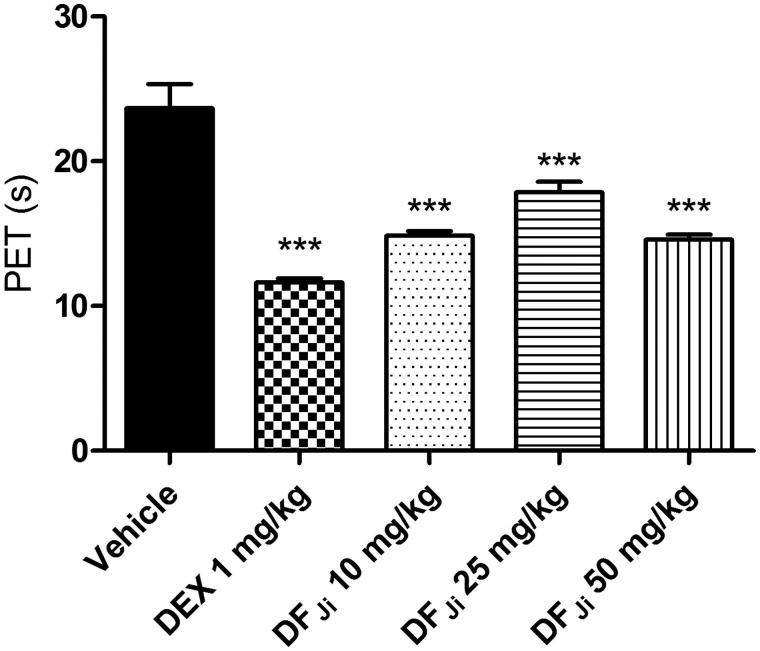
Effect of intravenous administration of the *J. isabellei* dichloromethane fraction (10, 25, and 50 mg/kg) and dexamethasone (DEX, 1 mg/kg) on paw elevation time (PET). The animals were treated 2 h after the intra-articular carrageenan injection (300 μg/knee). The negative control group received the vehicle (DMSO:polysorbate 80:saline solution 5:4:91). ***p < 0.001 represents a significant difference compared with the negative control group. The statistical analysis was performed using one-way ANOVA followed by Dunnett’s *post hoc* test.

By the intravenous route, the DF*_Ji_* also produced a significant reduction of incapacitation, but with nearly 4-fold lower doses. The average PET values were 14.8 ± 0.3 s, 17.8 ± 0.7 s and 14.5 ± 0.4 s (*p* < 0.001) for the doses of 10, 25 and 50 mg/kg, respectively (Figure 2). Vehicle-treated animals showed an average PET value of 23.7 ± 1.6 s. Intravenous administration of dexamethasone at a dose of 1 mg/kg (positive control group) caused a decrease of incapacitation (11.6 ± 0.3 s), similar to that produced after the DF*_Ji_* intravenous administration. The administration of the DF*_Ji_* at doses of 1 mg/kg and 0.1 mg/kg did not produce an effect on the PET (data not shown).

#### Effect of the oral administration of the DF_Ji_ on the rat paw edema

**Figure 3.**
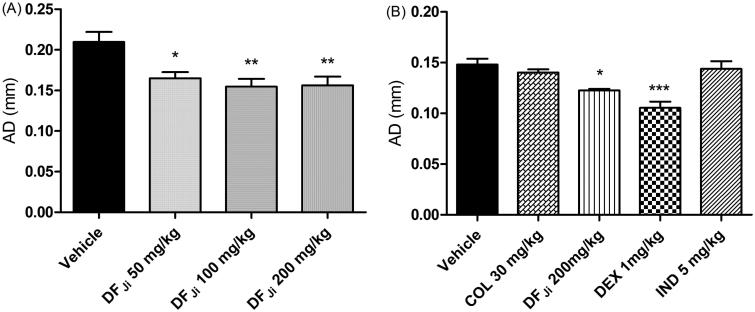
Effect of oral administration of the *J. isabellei* dichloromethane fraction (DF*_Ji_*) on the rat paw edema. (A) Articular diameter (AD) of animals receiving different doses of the DF*_Ji_*. (B) Articular diameter of animals receiving DF*_Ji_* (200 mg/kg) and the positive controls indomethacin (IND, 5 mg/kg), colchicine (COL, 30 mg/kg), and dexamethasone (DEX, 10 mg/kg). The animals were treated 2 h after the intra-articular carrageenan injection (300 μg/knee). The negative control group received the vehicle (DMSO:PEG 400:PBS 5:47.5:47.5). *p < 0.05, **p < 0.01 and ***p < 0.001 represent a significant difference compared with the negative control group on the day of the experiment. The statistical analysis was performed using one-way ANOVA followed by Dunnett’s *post hoc* test.

A single 300 μg intra-articular injection of carrageenan produced a progressive increase in the rat knee-joint diameter, indicating the development of edema. Oral treatment with the DF*_Ji_* partially prevented the edema. The doses of 50, 100 and 200 mg/kg produced a reduction in the AD of 0.045, 0.055 and 0.054 mm, when compared with the negative control group, which corresponded to a reduction of 21.3, 26.2 and 25.3%, respectively, in the articular edema (*p* < 0.05, 0.01 and 0.01, respectively) (Figure 3(A)). This edematogenic response was insensitive to orally administered indomethacin (5 mg/kg) and colchicine (30 mg/kg), while dexamethasone (10 mg/kg) was able to significantly reduce the articular diameter (*p* < 0.001). There was no statistical difference in the articular diameter between the groups that received the DF*_Ji_*and dexamethasone (Figure 3(B)).

#### Effect of the intravenous administration of the DF_Ji_ on the rat paw edema

**Figure 4.**
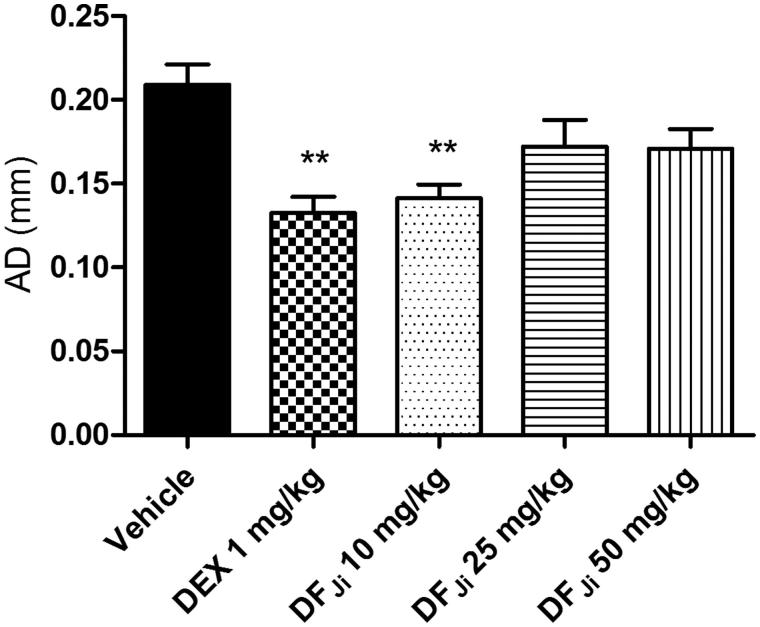
Effect of intravenous administration of the *J. isabellei* dichloromethane fraction (DF*_Ji_*) and dexamethasone (DEX, 1 mg/kg) on the rat paw edema. The animals were treated 2 h after the intra-articular carrageenan injection (300 μg/knee). The negative control group received the vehicle (DMSO:polysorbate 80:saline solution 5:4:91). **p < 0.01 represents a significant difference compared with the negative control group. The statistical analysis was performed using one-way ANOVA followed by Dunnett’s *post hoc* test.

By intravenous route, the DF*_Ji_*was able to prevent the increase of the articular diameter only at dose of 10 mg/kg, showing an decrease in the AD of 0.068 mm (32.5%) when compared with the negative control group (*p* < 0.01). No statistical difference was found in the paw diameter between the group receiving the DF*_Ji_* and the group receiving dexamethasone at doses of 10 and 1 mg/kg, respectively (Figure 4). Similarly to what was verified for the PET values, the intravenous administration of the DF*_Ji_* at doses of 1 and 0.1 mg/kg did not have an effect on edema (data not shown).

### Isolation and identification of the diterpene jatrophone from the DF_Ji_

**Figure 5.**
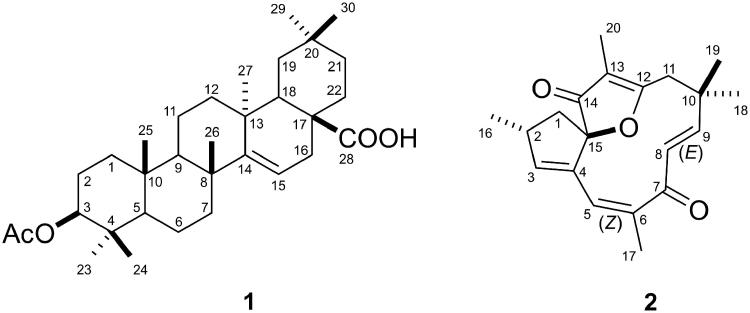
Structure of acetyl aleuritolic acid (**1**) and jatrophone (**2**) isolated from the DF*_Ji_*.

The dichloromethane fraction (1.05 g) was submitted to column chromatography and gave the sub-fractions 19–20 and 24–25 which, after precipitation with hexane, resulted in compounds **1** (2.4 mg) and **2** (5.0 mg), respectively (Figure 5).

Compound **1** was identified as acetyl aleuritolic acid by the ^13^C NMR (chloroform-*d*_6_, 400 MHz) assignments which are in agreement with data published by Fröhlich et al (2013). This triterpene was previously isolated from *J. isabellei* of combined petroleum ether and ethyl acetate fractions by Pertino et al. (2007b) and from the dichloromethane fraction by Fröhlich et al. (2013).

Compound **2** was identified as jatrophone and was obtained as colorless crystals. ^1^H NMR (acetone-*d*_6_, 600 MHz) assignments of jatrophone were as follows: *δ* H (ppm) 2.15 (1H, dd, *J* = 13.6; 5.9 Hz, H-1a), 1.74 (1H, dd, *J* = 13.6; 7.8 Hz, H-1b), 2.92–2.99 (1H, m, H-2), 5.68 (1H, m, H-3), 5.71(1H, m, H-5), 5.91 (1H, d, *J* = 16.3 Hz, H- 8), 6.58 (1H, d, *J* = 16.3 Hz, H-9), 3.04 (1H, d, *J* = 15.1 Hz, H-11a), 2.47 (1H, dd, *J* = 15.1; 0.8 Hz, H-11b), 1.08 (3H, d, *J* = 7.1 Hz, H-16), 1.83 (3H, d, *J* = 1.6 Hz, H-17), 1.25 (3H, s, H-18), 1.37 (3H, s, H-19) and 1.67 (3H, d, *J* = 0.8 Hz, H-20). ^13^C NMR (acetone-*d*_6_, 150 MHz) were as follows: *δ* C (ppm) 43.2 (C-1), 39.3 (C-2), 147.1 (C-3), 139.2 (C-4), 123.9 (C-5), 143.6 (C-6), 201.7 (C-7),129.4 (C-8), 160.3 (C-9), 37.5 (C-10), 41.6 (C-11), 184.4 (C-12), 113.1 (C-13), 203.6 (C-14), 100.3 (C-15), 19.7 (C-16), 21.1 (C-17), 30.6 (C-18), 27.4 (C-19) and 6.4 (C-20). The ^13^C NMR shifts as well as the 2D long-range heteronuclear correlations (HMBC) observed for compound **2** are in agreement with the data previously reported for the diterpene jatrophone (Goulart et al. 1993; Batista et al. 2014), although, recent studies have demonstrated results in which chemical shifts for carbons C-3 and C-5 of this compound were inverted (Fernandes et al. 2013; Sahidin et al. 2013). The mass spectrum indicated a molecular weight of 313.17980 *m/*z [M + H]^+^, with 0.06 ppm of error (Calcd for C_20_H_24_O_3_H^+^_,_ [M + H]^+^: 313.17982 *m/*z). The UV/VIS spectrum obtained in acetonitrile showed a *λ*_max_ of 280 nm.

### Development and validation of the UFLC-DAD method

**Figure 6.**
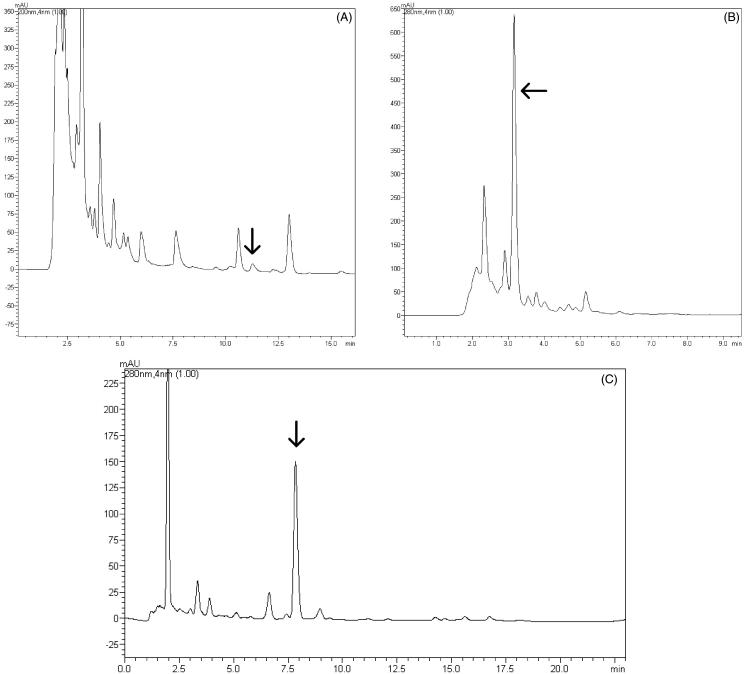
UFLC-DAD chromatograms obtained for the DF*_Ji_*. (A) Detection at 200 nm, indicating the acetyl aleuritolic acid, (b) detection at 280 nm, enlarged, indicating the diterpene jatrophone, (c) detection at 280 nm displaying the jatrophone compound with a good resolution.

For the development of the analytical method by UFLC-DAD, several conditions were previously tested, including different proportions of acetonitrile:water as eluent and flow rates from 0.8 to 1.0 mL/min. A first separation of the terpenic compounds was carried out using a C18 column and a mobile phase gradient starting with 90% of acetonitrile and 10% of water, followed by an isocratic condition with 100% of acetonitrile with a flow rate of 0.8 mL/min. Using these conditions, it was possible to separate acetyl aleuritolic acid, which showed retention time of 10.44 min (Figure 6(A)). The presence of acetyl aleuritolic was verified by comparing the retention times and by spiking the sample with the triterpene before the UFLC analysis. On the other hand, the presence of a large peak displaying retention time of 3.06 min was visualized in this UFLC chromatogram, when the detector was set at 280 nm (Figure 6(B)). The identification of this compound was performed by mass analysis in a mass spectrophotometer (Bruker micro TOF-QII, source type APPI, operating in positive mode) coupled to the LC system. The result indicated that this peak corresponded to the diterpene jatrophone. Since it appeared to be an important compound of this fraction, the chromatographic conditions were changed in order to obtain a better resolution of this peak and use this compound as a possible chemical marker of the DF*_Ji_*. To obtain a better separation of jatrophone, the mobile phase was eluted at flow rate of 1.0 mL/min in a gradient mode starting from 53:47 acetonitrile:water, which was increased to 65% acetonitrile over 12 min and then to 75% acetonitrile over 15 min of analysis. In such conditions, the total run time was 23 min and jatrophone retention time was 7.8 min (Figure 6(C)).

The method developed to quantify jatrophone in the DF*_Ji_* was validated according to the ICH and the ANVISA guidelines (Brazil 2003; ICH 2005). The calibration curve (*y* = 41.010 × –270.76) obtained for jatrophone showed a correlation coefficient (r^2^) of 0.9997, indicating that the method is linear over the concentration range from 1.0 to 100.0 μg/mL. The lowest amount of jatrophone, which could be detected (LOD) and quantitatively determined (LOQ), was 0.04 and 0.15 μg/mL, respectively. Measurements of intra- and inter-day were used to determine the precision of the method and evaluated by the relative standard deviation (RSD%). The intra-day and inter-day precision analyses indicated RSD of 1.73% and 1.94%, respectively. The accuracy of the UFLC method was assessed by the recovery data. The recovery values obtained for the jatrophone-spiked dichloromethane fraction were higher than 90% for the three levels evaluated (Table 1), and this result can be considered acceptable for the analysis of the compounds in a complex matrix as a plant extract. The peak purity index found was 0.999948, indicating that jatrophone is clearly separated from any interfering peak, demonstrating the specificity of the developed method (ICH 2005).

**Table 1.** Recovery values (%) obtained for the evaluation of the accuracy of the method.

Constituent	Spiked (μg/mL)	Found (μg/mL) (SD)^a^	Recovery (%)
Jatrophone	5.45	5.86 (0.20)	107.65
	27.25	26.19 (0.09)	96.13
	81.75	81.38 (0.43)	99.54

a *n* = 3, triplicate injection.

Considering the results described above, the UFLC method was found to be linear, specific, precise, and accurate to determine jatrophone in the *Jatropha isabellei* dichloromethane fraction. After the analysis, the results indicated the presence of 89.68 ± 1.55 μg of jatrophone per milligram of extract, which correspond to a concentration of 8.97% (w/w) of this diterpene in the dichloromethane fraction.

## Discussion

In this study, the effects of the orally and intravenously administered dichloromethane fraction from *J isabellei* (DF*_Ji_*) for articular pain and edema were evaluated using the hind paw elevation time and knee diameter of rats sensitized with an intra-articular injection of carrageenan. The dichloromethane fraction reduced both incapacitation and articular edema after oral and intravenous administration, indicating that the chemical constituents in this fraction demonstrated antinociceptive and antiedematogenic properties. Only the DF*_Ji_*and dexamethasone were effective in reducing both parameters by both administration routes.

Previous studies have addressed the likely anti-inflammatory and antinociceptive mechanisms of the terpenoid constituents found in the DF*_Ji._* The diterpene jatrophone, which may be considered as a possible chemical marker of the DF*_Ji_*, has previously shown to inhibit lymphocyte proliferation, presumably through inhibition of the protein kinase C (PKC) pathway, which in turn mediates a number of intracellular signaling pathways involved in the pathogenesis of inflammation (Moraes et al. 1996). Other constituents previously isolated from the DF*_Ji_*, as sitosterol and acetyl aleuritolic acid, also have demonstrated an anti-inflammatory effect on edema induced by carrageenan (Perazzo et al. 2007; Bhalke & Pal 2012). On the other hand, the lack of an antiedematogenic effect with the higher doses of DF*_Ji_*after intravenous administration may be explained, at least in part, by a vasodilating effect of jatrophone. The vasodilating effect of this drug was demonstrated in the portal vein and aorta of the rats and it was attributed to the inhibition of a PKC-dependent mechanism (Silva et al. 1995), as well as the inhibition of Ca^2+ ^influx and activation of K^+ ^channels (Duarte et al. 1992). This vasodilation could be attenuating its antiedematogenic effect. Furthermore, jatrophone was able to inhibit the [^3^H]glutamate binding in a dose-dependent way, which also supported an antinociceptive effect of this compound (Martini et al. 2000).

The effective oral dose of the DF*_Ji_* was about 20 and 5 times higher than the effective intravenous dose for the antinociceptive and antiedematogenic effect, respectively. These findings are supposed to be due to the incomplete absorption of the chemical constituents of the dichloromethane fraction after oral administration. Many factors affect the oral absorption of drugs, including the anatomical and physiological characteristics of the gastrointestinal tract and the physicochemical properties of the drug. In this case, the lower pharmacological effectiveness of the orally administered DF*_Ji_* could be explained in part by the low water solubility of the terpenes and phytosterols present in this fraction (Rossi et al. 2010; Thoppil & Bishayee 2011; Duchateau et al. 2012). Thus, formulations that allow the increase of the solubility of the chemical constituents of the DF*_Ji_* in the biologic fluids should be developed to overcome their limited oral absorption and to take advantage of the beneficial pharmacological properties of this herbal medicine to treat arthritis.

The selection of chemical markers is very important for the quality control of herbal medicines since their content should be determined at various stages of the development and manufacturing of a product. Some of these stages comprise authentication and differentiation of species, harvest of the best quality raw materials, quality evaluation of intermediates and finished products, stability assessment, and detection of toxic compounds (Li et al. 2008). The diterpene jatrophone was isolated from the DF*_Ji_* and quantified by UFLC-DAD method. Because a large amount of this compound was found in this fraction and considering its contribution for the anti-arthritic activity, it was supposed that jatrophone could be used as a chemical marker of the DF*_Ji_*. Additionally, this diterpene could be determined by using a UFLC-DAD method, in which the chromatographic conditions allowed to obtain a satisfactory separation of the jatrophone peak from the other constituents of the dichloromethane fraction in a short time and with minimal tail. The chromatograms exhibited a good baseline resolution and the method was successfully validated, according to the results presented above. Taking into account the results obtained in this study, the development of new dosage forms from the *J. isabellei* dichloromethane fraction may be considered as promising research to be undertaken.

## Conclusions

The dichloromethane fraction from *J. isabellei* was able to produce antinociceptive and antiedematogenic effects in a model of acute arthritis in rats, when administered by both oral and intravenous routes. This effect may be related to the presence of terpenes in this fraction, especially the diterpene jatrophone. The pharmacological effectiveness was higher after intravenous administration, indicating that the chemical compounds from DF*_Ji_* exhibits limited absorption in the gastrointestinal tract. The UFLC-DAD analytical methodology developed in this study allowed to quantify the jatrophone in the DF*_Ji_*, which was suggested to be used as a chemical marker to guide the development of drug dosage forms from this fraction. Thus, the DF*_Ji_*may represent a promising plant product for the development of herbal medicines for the treatment of arthritis.
